# Conversational hypnosis versus standard of care to reduce anxiety in patients undergoing marker placement under radiographic control prior to breast cancer surgery: A randomized, multicenter trial

**DOI:** 10.3389/fpsyg.2022.971232

**Published:** 2022-11-22

**Authors:** Lydie Lemoine, Virginie Adam, Xavier Galus, Pascale Siles, Agnès Coulon, Jeannette Grenier-Desforges, Joseph Orabona, Isabelle Kergastel, Pierre Wagner, Julia Salleron, Priscillia Tosti, Cécile Huin-Schohn, Jean-Louis Merlin, Rémi Etienne, Philippe Henrot

**Affiliations:** ^1^Department of Radiology, Institut de Cancérologie de Lorraine, Vandoeuvre-lès-Nancy, France; ^2^Department of Supportive Care in Oncology, Institut de Cancérologie de Lorraine, Vandoeuvre-lès-Nancy, France; ^3^Department of Radiology, Centre Hospitalier Universitaire la Timone, Marseille, France; ^4^Department of Radiology, Centre Léon Bérard, Lyon, France; ^5^Department of Radiology Centre Aliénor d’Aquitaine-CHU Pellegrin, Bordeaux, France; ^6^Department of Radiology, Centre Hospitalier de Bastia, Institut du Sein, Bastia, France; ^7^Department of Radiology, Centre Hospitalier Universitaire de Brest, Brest, France; ^8^Department of Radiology, Centre Paul Strauss, Strasbourg, France; ^9^Departement of Biostatistics, Institut de Cancérologie de Lorraine, Vandoeuvre-lès-Nancy, France; ^10^Departement of Clinical Research, Institut de Cancérologie de Lorraine, Université de Lorraine, Vandoeuvre-lès-Nancy, France

**Keywords:** conversational hypnosis, breast cancer, surgery, anxiety, radiology

## Abstract

**Background:**

Surgery is a cornerstone of breast cancer management. Prior to surgery, a wire marker is placed at the site of the tumor, to enable the surgeon to accurately localize the lesion during later surgery. This procedure can generate considerable anxiety for many patients. We investigated the value of conversational hypnosis (CH) in reducing anxiety in patients undergoing preoperative wire placement under radiographic control.

**Methods:**

Randomized, multicentre study in 7 centers in France. Inclusion criteria were patients aged >18 years with an Eastern Cooperative Oncology Group performance status ≤2, scheduled to undergo preoperative wire placement in one or several breast lesions. Patients were randomized in a 1:1 ratio, stratified by center to undergo preoperative wire placement with or without the use of CH by a radiological technician trained in the CH technique. The primary endpoint was the percentage of patients with an anxiety score ≥ 6 on a visual analog scale ranging from 0 (absence of anxiety) to 10 (maximal anxiety). Secondary endpoints were pain score, perceived duration reported by the patient, technician satisfaction with their relationship with the patient, and ease of marker insertion reported by the radiologist. Semi-structured interviews were performed with patients to assess their perception of the marker placement procedure.

**Results:**

The trial was prematurely interrupted for futility after a planned interim analysis after accrual of 167 patients, i.e., half the planned sample size. Prior to marker placement, 29.3% (*n* = 24) of patients in the control group had an anxiety score ≥ 6, versus 42.3% (*n* = 33) in the CH group (*p* = 0.08). After marker placement, the change of anxiety score was not significantly different between groups (11.0% (*n* = 9) versus 14.3% (*n* = 11), *p* = 0.615). There was no significant difference in any of the secondary endpoints. In the interviews, patients from both groups frequently spoke of a feeling of trust.

**Conclusion:**

This study failed to show a benefit of conversational hypnosis on anxiety in patients undergoing marker placement prior to surgery for breast cancer. The fact that some caregivers had learned this personalized therapeutic communication technique may have had a positive impact on the whole caregiving team.

**Trial registration:**

The study was registered with ClinicalTrials.gov (NCT02867644).

## Introduction

With approximately 58,500 new cases in France in the year 2018, breast cancer remains one of the most common female cancers, and the risk increases with age (Defossez et al., 2021). Surgery remains the cornerstone of therapy, with a view to complete removal of the tumor, thereby enabling analysis of its size, aggressiveness, and the expression of hormone receptors. These findings are key to orienting therapy. Prior to surgery, a wire marker is placed at the site of the tumor under radiographic control, to enable the surgeon to accurately localize the exact site during subsequent surgery. The radiologist inserts a needle into the abnormal area or at the site of the clip placed during biopsy. A small thin wire is inserted, and the needle is removed, leaving the wire in place to indicate the exact site of the tumor.

The announcement of a breast cancer diagnosis can cause a great deal of anxiety and even depression, as it has a profound impact on physical appearance, sexual identity, and life expectancy, amongst other aspects, and can make real to patients the idea of their own death (Boivin et al., 2021). Furthermore, the wire placement, although minimally invasive, may be painful, or cause anxiety for many patients (Kelly and Winslow, 1996). This anxiety may be multifactorial, caused by the procedure itself, the fear of the subsequent surgery, and may also be influenced by prior painful experiences.

For several years now, the value of hypnosis has been demonstrated in patients with breast cancer (Grégoire et al., 2018). A systematic review performed in 2014 (Cramer et al., 2015) reported rare but promising findings regarding the efficacy of hypnosis in reducing pain and distress in women with breast cancer or undergoing diagnostic breast biopsy. Numerous other studies published since then have also reported the utility of hypnosis during breast cancer management (Téllez et al., 2020; Berliere et al., 2021), especially during the perioperative period (Potie et al., 2016). In the multicentre, randomized HYPNOSEIN study (Amraoui et al., 2018), which investigated the effect of hypnosis before general anesthesia, hypnosis was associated with significantly lower anxiety and fatigue after minor breast cancer surgery.

The hypnotic state leads an individual to experience a modified state of consciousness that is neither sleep, nor a waking state, but rather a state of focused attention and heightened awareness. Various methods exist for practicing hypnosis (Short, 2018; Etienne et al., 2021). Among them, conversational hypnosis (CH) has not been widely studied in clinical research (Izanloo et al., 2015; Bataille et al., 2017; Boselli et al., 2018; Sourzac et al., 2018). This approach aims to distract the patient’s attention away from a potentially painful experience. The light “trance” state induced by hypnosis makes it possible to “dissociate” the patient’s mind and thereby reduce the moral or physical discomfort. The work of Michaux et al. (2007) underlines that the originality of CH relies on two key aspects, namely the communication tools that enable a strong but nondirective relation with the patient, and second, the therapeutic principle of CH. This latter is based on the assumption that the patient possesses the inner resources to respond appropriately to the situations he/she may encounter, and consequently, CH is about mobilizing these personal competences and capacities to adapt (Michaux et al., 2007).

Against this background, and in the specific context of anxiety generated by an interventional procedure and the fear of subsequent surgery, we performed a randomized, multicentre study to investigate the benefit of CH compared to standard management, in reducing anxiety in patients undergoing preoperative wire placement under radiographic control. Secondary objectives were to compare patient-reported pain levels, overall perception of the patient about the wire placement procedure, ease of the procedure for radiologist and the caregiver’s satisfaction regarding their relationship with the patient during the procedure.

## Materials and methods

### Study design

We performed a prospective, randomized, single-blind, multicentre study in 7 centres in France (Institut de Cancérologie de Lorraine (Nancy), Centre Paul Strauss (Strasbourg), Groupe hospitalier Pellegrin (Bordeaux), Hôpital de la Timone (Marseille), Centre Léon Bérard (Lyon), the University Hospital of Brest and the University Hospital of Bastia). The study was registered with ClinicalTrials.gov under the number NCT02867644, and was approved by the Ethics Committee CPP (Comité de Protection des Personnes) Est III on 3 May 2016 under the number ID-RCB: 2016-A00232-49, and by the French Agency for Health Products Safety (ANSM) on 22 April 2016 under the number 160225B-31. All patients were informed about the study and all provided written informed consent. The study was financed by the national hospital nurse & paramedical research programme (Programme Hospitalier de Recherche Infirmière et Paramédicale—PHRIP; grant obtained in 2015). The study was performed in accordance with the Declaration of Helsinki and is reported following the international CONSORT guidelines (Consolidated Standards of Reporting Trials).

### Study population

Inclusion criteria were patients aged > 18 years with an Eastern Cooperative Oncology Group (ECOG) performance status ≤ 2, and scheduled to undergo preoperative wire placement in one or several breast lesions under radiographic control. Exclusion criteria were patients with hearing impairment, patients suffering from schizophrenia, patients who were unable to understand French, pregnant or breastfeeding women, and women under legal or judicial protection.

### Randomization and blinding procedure

The study was proposed to patients with a breast lesion who were scheduled to undergo preoperative wire placement. Patients were informed that the objective of the study was the comparison of two groups: (1) preoperative wire placement using the standard procedure (control group) and (2) preoperative wire placement with CH (hypnosis group). After providing informed consent, patients were randomized on an equal basis between two groups (i.e.1:1 ratio). Randomization was centralized by computer-generated random numbers in blocks of 4, with stratification by center, using CleanWeb^©^ software. Allocation concealment mechanism was based on a randomization list created before the start of the trial. The list was thus used when a patient was included into the trial (*via* Cleanweb) and the result of this randomization was available on Cleanweb. CH was performed by radiological technicians trained in the use of this method of communication. To reduce cross-contamination bias, patients from the control group were to be managed by radiological technicians who were not trained in CH, while those in the hypnosis group were to be managed by radiological technicians trained in the use of CH. Patients were unaware of their randomization group (single-blind) until the end of the study.

### Description of the hypnosis intervention

In the control group, as soon as the patient was set up in the examination room, the radiological technician prepared the patient for the exam and explained how the procedure would be performed. In the hypnosis group, the same explanations were given using CH during the communication. In the control group, the radiologist was informed as soon as the patient was ready, and could then begin the ultrasound examination. When the radiologist had identified the target zone, the technician and radiologist prepared the material for the wire placement, and prepared the patient according to usual practice. The radiologist placed the markers, and once finished, the radiological technician dressed the patient’s wound and accompanied the patient back to the waiting room, in accordance with the local practice in each center. In the hypnosis group, the same procedure was performed but throughout the whole duration of the procedure, the radiological technician communicated with the patient using the CH technique (according to the methods learned during training). The intervention manual is available in Supplementary material.

For both groups, the radiologist was aware of the patient’s randomized group and therefore refrained from participating in the conversation.

The radiological technicians who were trained in CH received two days of training (Teleska and Roffman, 2004) in this technique from a qualified expert from the Hypnosis Training Institute (Ipnosia Nancy, France). The training covered hypnotic communication between the patient and hypnotherapist, specifically during radiographic preoperative procedures for marker placement. The training covered the theoretical aspects of the discipline of hypnosis, while also adapting to the practical aspects linked to the procedure. The content of the training programme is detailed in the Supplementary Material.

### Data recorded

Anxiety was evaluated in both groups on a visual analog scale (VAS), ranging from 0 to 10 (Millar et al., 1995; Rossi and Pourtois, 2012). Anxiety was measured immediately prior to the procedure (as soon as the patient was set up in the examination room), and immediately after marker placement, with 0 corresponding to the absence of anxiety, and 10 to maximal anxiety. The primary endpoint of the study was the percentage of patients in each group with a score of 6 or more on the anxiety scale immediately after marker placement (Lang et al., 2006). Patient anxiety was also measured using the State–Trait Anxiety Inventory (STAI) Y-A form (state anxiety) when the patient was in the waiting room before and after marker placement. The STAI-Y-A form refers to how the patient feels “right now, that is, at this moment,” and the score ranges from 20 to 80, as follows: (1) severe anxiety (≥ 66); (2) high anxiety (56–65); (3) moderate anxiety (46–55); (4) mild anxiety (36–45); and (5) very mild anxiety (≤ 35). As for anxiety, pain was also evaluated using a VAS, immediately before and after marker placement. A score of 0 corresponds to the absence of pain, and 10 to maximum pain.

The total duration of the procedure (from entry into the examination room until the end of marker placement) was measured using a chronometer. The duration of the procedure, as perceived by the patient, from the time of entry into the examination room to the end of marker placement, was estimated by the patient. This enabled us to evaluate temporal distortion (which is one of the characteristics of the hypnotic trance) by calculating the difference between the actual duration and the duration perceived by the patient.

**Figure 1:**
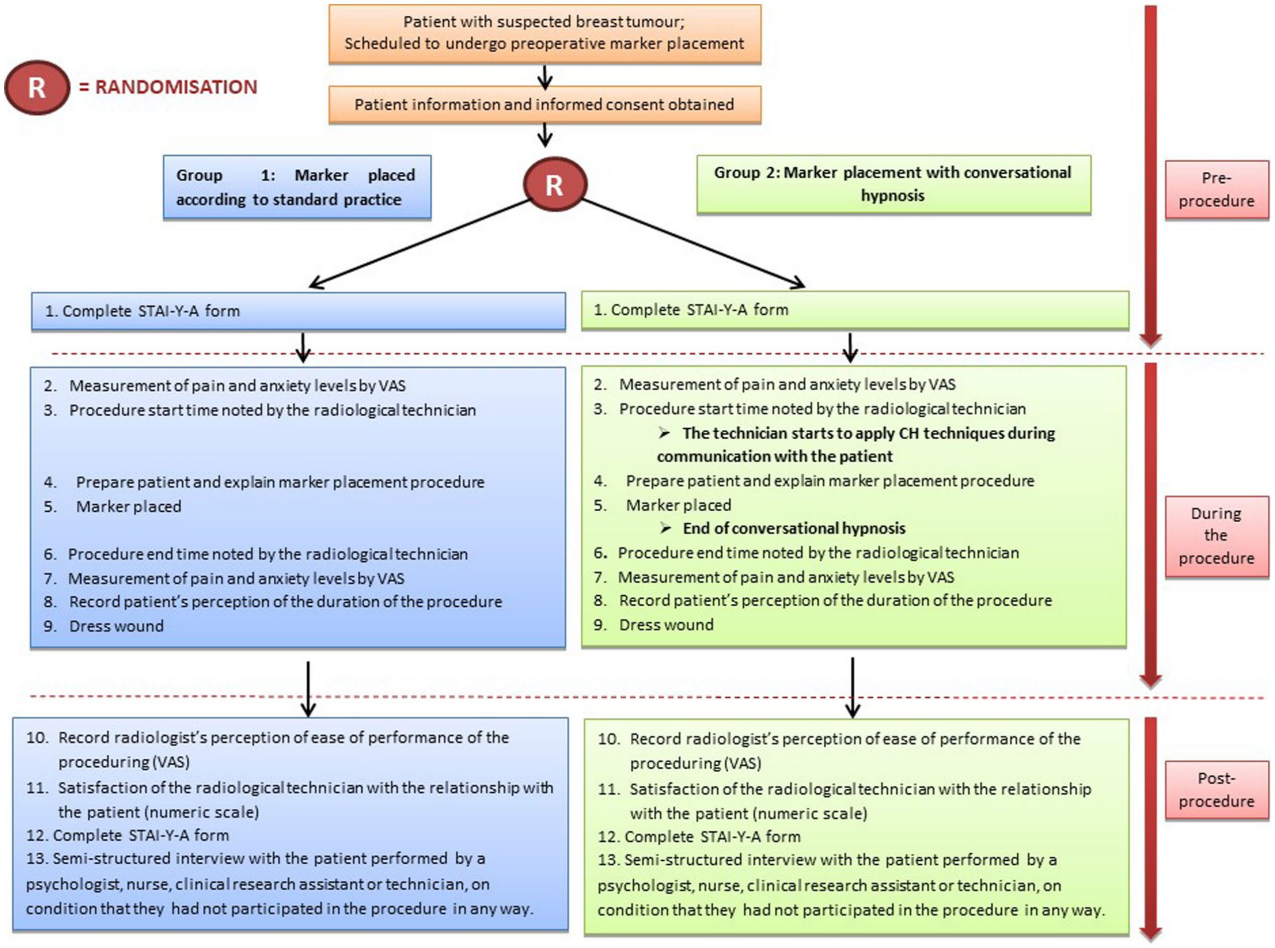
Study design.

The radiological technician’s satisfaction with their relationship with the patient during the procedure was evaluated by a numeric scale at the end of the procedure (with 0 corresponding to an excellent relationship with the patient, and 10 to a very poor relationship). The radiologist evaluated the ease of insertion of the marker using a VAS (with 0 corresponding to very easy, and 10 corresponding to very difficult). The outline of the study procedure and measurements is illustrated in Figure 1.

When the patient returned to the waiting room after the procedure, a semi-structured interview was performed with her to assess her perception of the marker placement procedure. The interview was performed by a psychologist, nurse or radiological technician, the only eligibility criterion being that the interviewer must not have participated in the procedure in any way. The interview was audio-recorded and explored four points, namely the patient’s anticipated representation of the procedure, their physical and emotional sensations during the procedure, and the memory left by the procedure. Finally, the randomization group was revealed to the patient by the interviewer.

Data were publicly available *via* an open repository at https://osf.io/8e4xd/.

### Statistical analysis

Before the start of the study, we hypothesized that after the procedure, 40% of patients in the control group would have an anxiety score of 6 or more, versus 25% in the hypnosis group (Lang et al., 2006). With an alpha risk of 5 and 80% power, a total of 152 patients per group were required. Allowing for 10% of patients unsuitable for analysis, a final total of 167 patients per group was required. An interim analysis was planned after accrual of 50% of the patients. To avoid inflation of the alpha risk, the level of significance for the interim analysis was set at 0.001 according to the method of Peto et al. (1976). The study could be discontinued for efficacy if, after the interim analysis, a significant difference at the 0.001 level was observed in the primary endpoint of anxiety felt by the patients after the procedure.

Analyses were performed by intention-to-treat based on the initial treatment assignment and not on the treatment actually received. For the primary outcome, the percentage of patients with an anxiety score of 6 or more was compared between groups using the chi square test. The scores after marker placement were assessed using a generalized mixed model with a logit link. The numerical value of the anxiety score from the VAS was also compared before the marker placement with a Student *T*-test or a Mann–Whitney *U* test according to the normality of the data, as assessed by the values of skewness and kurtosis. The VAS scores for anxiety after marker placement were compared between groups using a linear mixed model. The normality of residuals was investigated as well as the leverage effect and Cook’s distance. When the normality of residuals was not stratified, a Box–Cox transformation was performed by choosing the optimal power parameter according to the maximum likelihood criterion. For these mixed models, the within-subject correlation was modelized with a compound-symmetry covariance matrix. The same analyses were performed for the VAS pain score, the STAI Y-A score, and the duration of the procedure. Radiologist-reported scores regarding the ease of performance of the procedure and the technician-reported satisfaction scores were compared between groups using the Student t test or the Mann–Whitney U test. All analyses were performed using SAS version 9.4 (SAS Institute Inc., Cary, NC, United States). A value of *p* < 0.001 was considered statistically significant. SAS code for the main outcomes is publicly available *via* an open repository at https://osf.io/8e4xd/. Qualitative analysis of the semi-structured interviews was performed with the aid of the text analysis software Tropes (a free software programme developed by P. Molette and A. Landré based on work by R. Ghiglione).^1^ Audio-recorded interviews were fully transcribed. Our study was based on conventional content analysis technique (Vaismoradi et al., 2013). The interviews were analyzed separately for each group and blinded to trial allocation. To ensure the robustness of analyses, our study was triangulated data in two ways: data source triangulation for the transcription (VA and RE) and triangulation to compare alternative interpretations and reveal any inconsistencies (VA and RE) (Patton, 1999).

## Results

**Figure 2:**
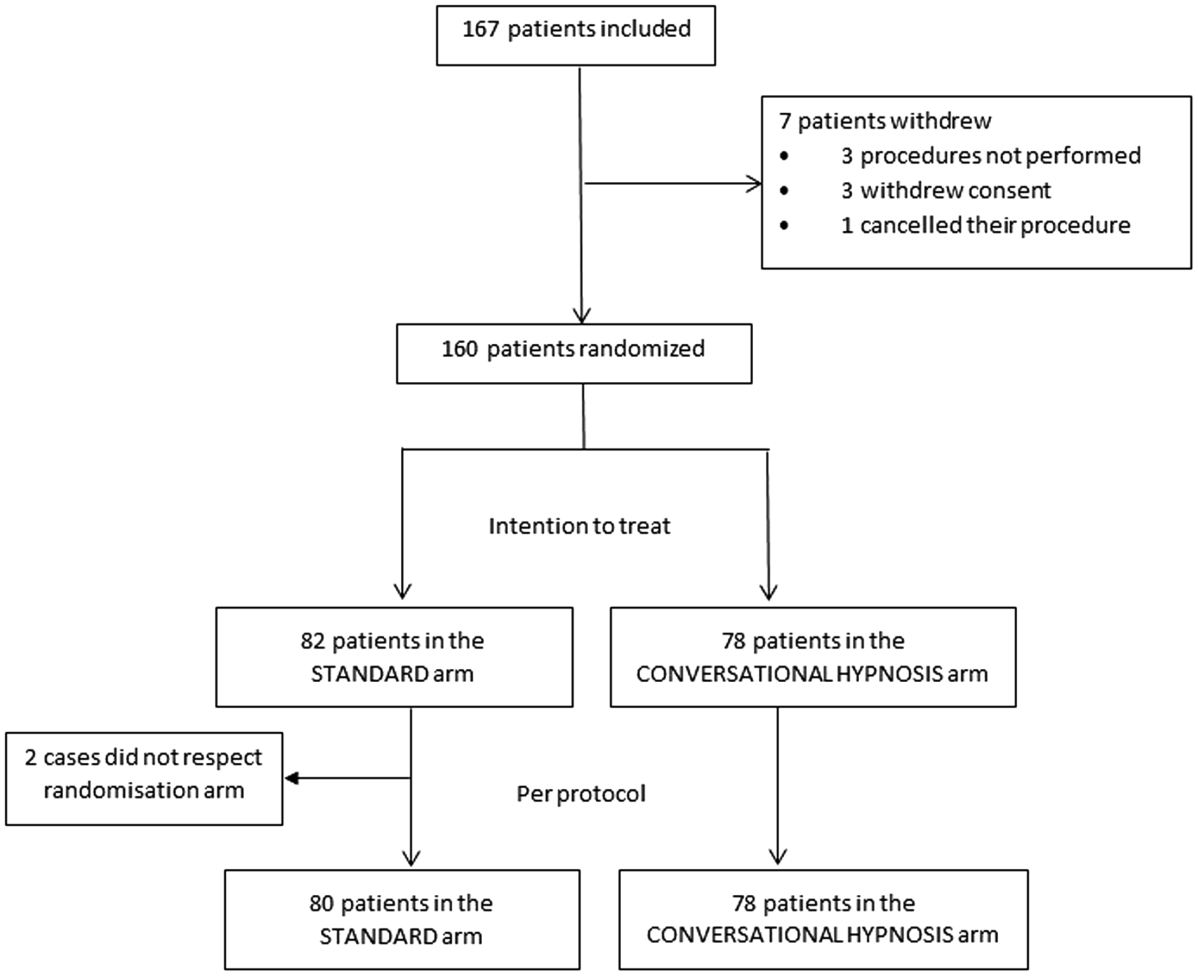
Flow-chart.

The trial was prematurely interrupted for futility after the planned interim analysis of data from 167 patients included between 17 November 2016 and 25 June 2020. Seven patients had withdrawn from the study prematurely, and a total of 160 patients were randomized; 82 to the control group, and 78 to the hypnosis group (Figure 2). The imbalance between the group sizes was due to the block randomization size and the stratification by center. Two patients who were randomized to the control group actually received CH.

The baseline characteristics of the patients are presented in Table 1. In the control group, 21 patients (32.3%) underwent their procedure with a radiological technician trained in CH, but without receiving CH during the procedure.

**Table 1:** Characteristics of the patients randomized to the control and conversational hypnosis arms.

	Standard *N* = 82	Conversational hypnosis *N* = 78
Age, mean ± SD (min–max)	59.3 ± 10.7 (21–85)	61.4 ± 9.9 (29–88)
Number of lesions^◊^, % (*n*) 1 2 4	92.4% (73) 6.3% (5) 1.3% (1)	89.5% (68) 10.5% (8) 0% (0)
1	92.4% (73)	89.5% (68)
2	6.3% (5)	10.5% (8)
4	1.3% (1)	0% (0)
Technique used^*^, % (*n*)		
Metal wire	98.8% (81)	100% (76)
Biopsy	1.2% (1)	0
Local anesthetic^∆^, % (*n*)	39.0% (32)	39.0% (30)
Technician trained in hypnosis^‡^, %(*n*)	32.3% (21)	100% (77)

SD, standard deviation.

^◊^5 missing data; *2 missing data; ^∆^1 missing data; ^‡^18 missing data.

Prior to marker placement, 29.3% (*n* = 24) of patients in the control group had an anxiety score of 6 or more as assessed by VAS, versus 42.3% (*n* = 33) in the hypnosis group (*p* = 0.08). The change of anxiety score after marker placement was not significantly different between groups (11.0% (*n* = 9) in the control group with a score of 6 or more, versus 14.3% (*n* = 11) in the hypnosis group, *p* = 0.615). Given the non-significant result and the slow accrual, the study was discontinued after the interim analysis.

The results of the secondary endpoints are displayed in Table 2. Anxiety measured by the STAI Y-A form was not significantly different between groups before the placement of the wire marker (*p* = 0.15). Its change between groups was also not significantly different (*p* = 0.146). There was no significant difference between groups in terms of pain, perceived duration of the procedure, ease of insertion of the marker as reported by the radiologist, or in the technician’s satisfaction with their relationship with the patient.

**Table 2:** Comparison of anxiety, pain, perceived duration of the procedure, ease of insertion of the marker as reported by the radiologist, and satisfaction of the technician with their relationship with the patient, according to randomization group.

	Standard *N* = 82	Conversational Hypnosis *N* = 78	Value of *p*
**STAI Y-A score** ^a^			
Before marker placement	43.0; 45.0 ± 13.3	46.0; 48.3 ± 15.3	0.150^d^
After marker placement	33.0; 34.6 ± 11.1	34.0; 35.3 ± 12.1	0.146^e^
Difference After-Before	−8.0; −10.3 ± 10.5	−11.0; −12.9 ± 11.7	
Anxiety^b^			
Before marker placement	4.0; 4.5 ± 2.5	5.0; 4.8 ± 3.0	0.466^d^
After marker placement	2.0; 2.6 ± 2.4	2.0; 2.5 ± 2.2	0.307^e^
Difference After-Before	−1.3; −1.9 ± 2.8	−2.0; −2.3 ± 2.6	
Pain^b^			
Before marker placement	0; 0.8 ± 1.9	0; 1 ± 2.2	0.552^f^
After marker placement	1.0; 1.6 ± 2.1	1.0; 1.9 ± 2.3	0.825^e,g^
Difference after–before	0; 0.8 ± 2.1	0; 0.9 ± 2.7	
Duration of the procedure (minutes)			
Actual	9.0; 10.4 ± 5.2	10.0; 11.1 ± 5.2	0.267^f^
Perceived	10 0.0; 9.3 ± 5.5	10.0; 10.1 ± 6.1	0.857^e,h^
Difference actual—perceived	1.0; 1.2 ± 7.2	1.0; 1.0 ± 7.1	
Ease of procedure for radiologist ^b^	1.0; 2.0 ± 2.2	2.0; 2.1 ± 2.0 0;	0.513^f^
Satisfaction of technician with relationship with patient ^c^	1.0;2.1 ± 2.7	1.9 ± 2.8	0.530^f^

Results were presented with median; mean ± standard deviation.

^a^Score STAY Y-A: range from 20 to 80. ^b^Visual analog scale from 0 (no anxiety/no pain/easy procedure) to 10 (maximum anxiety/maximum pain/procedure difficult). ^c^Visual analog scale from 0 (good relationship) to 10 (difficult relationship). ^d^Student *T*-test. ^e^Linear mixed model for the comparison of the evolution between groups. ^g^Test performed after Box-Cox transformation with lambda=-1.25. ^f^Mann–Whitney U test. ^g^Test performed after Box-Cox transformation with lambda=0.25.

Data from the semi-structured interview was available for 159 patients (77 from the control group and 78 from the hypnosis group). In both arms, the procedure mostly went better than the patients had imagined. For the others, the procedure was either in line with what they had imagined, or they had had no expectations regarding how the procedure would go. The information provided seemed to have enabled the patients to accurately anticipate how the procedure would go, and the absence of prior information increased the patient’s apprehension. In both groups, patients mainly reported a painful sensation, but of tolerable intensity (described by terms such as “minimal,” “small,” “light”). Other patients reported that they had not felt anything. Patients in the hypnosis group more frequently described a sensation of time “flying by,” or the procedure lasting “not long at all,” indicating that they perceived the duration to be shorter. Regarding their internal emotional feelings, patients from both groups frequently spoke of a feeling of trust. Factors that made them feel reassured during the procedure included the presence of other people in the room, smiles, and communication with the caregivers, either to explain things to them or to distract their attention. For those who reported a more stressful experience, they were nonetheless able to manage their stress. Regardless of the randomization group, numerous patients reported a contrast between before and after the procedure, whereby before the procedure, they were anxious worrying about it, then the actual experience resolved the anxiety for some participants (the “during”), while afterwards, many felt relief. Regarding the memory left by the procedure, patients from both groups predominantly described the experience using double-negatives (e.g., “it’s not a bad memory,” “it wasn’t very traumatic”). The main reasons cited were the absence or minimization of the pain they felt. Some patients even said they felt that they would be able to reassure other patients undergoing the same procedure, if needed. The patients from the hypnosis group especially underlined the soothing effects of the therapeutic relationship and accompaniment, using terms such as “trust,” “empathy,” “kindness,” “soothing environment,” or “bubble” that left “a good memory.”

## Discussion

The results of the interim analysis and the slow recruitment prompted the premature discontinuation of this randomized, multicenter trial. Contrary to the initial hypothesis, CH used during preoperative procedures for wire marker placement prior to breast cancer surgery did not significantly reduce patient anxiety compared to standard management. The level of pain experienced was also similar between groups.

The term “conversational hypnosis” should be interpreted in this study as a form of communication inspired by, or loosely based on hypnosis. Some American authors, such as Elvira Lang, use the term “comfort talk” (Zhang et al., 2021). The aims of these communication routines are to minimize pain and anxiety in the patient, by limiting the use of terms with negative connotations, by paying special attention to posture and how the patient is positioned, by favoring empathetic and interactive exchange with the patient, while simultaneously using indirect suggestion, as habitually employed during formal hypnosis (confusion, illusion of choice, yes sets, distraction, etc.). The protocol did not include a specific formal induction. Indeed, the aim of the study was precisely to distinguish the effects of a formal hypnosis session from conversation based on conversational hypnosis techniques. The only feature that resembled a form of focalization was the moment when the radiological technician asked the patient to describe their safe place, by asking for sensory details. This approach was mainly aimed at distracting the patient’s attention during the most unpleasant moments of the procedure. Consequently, the temporal distortion that is a hallmark of the hypnotic trance, and which corresponds to the difference between the actual duration of the procedure, and the length of time elapsed as perceived by the patient, was not significantly modified in the hypnosis arm of this study. However, during the semi-structured interviews performed with the patients after the procedure, the features of the hypnotic trance were frequently mentioned by the patients, even during CH, with those in the hypnosis group underlining their perception that the procedure was shorter than expected.

Anxiety and its management are key factors in determining quality of life among breast cancer patients (Gandhi and Oakley, 2005). The CH technique as applied in this study has not been widely assessed compared to formal hypnosis. We chose to focus on the procedure for wire placement prior to surgery because it is considered by the healthcare providers to be a major source of anxiety for patients, even though it only represents one small step in the overall management pathway. The design of the present study required the radiologist to refrain from participating in the hypnotic conversation, so as to avoid disturbing the trance state induced by this technique. Some radiologists may have felt that this “excluded” them, going against their usual practice. This may have partially explained the difficulties with patient recruitment, because the initial recruitment period was planned to be 24 months, whereas it took almost 4 years to accrue half the planned sample size.

The surprising results observed here may be explained by the fact that the anxiety felt before the procedure was actually lower than we had initially hypothesized. One of the possible reasons may be the use of the label hypnosis (Bioy et al., 2013). Prior to the procedure, 35% of randomized patients had a VAS score of 6 or higher, whereas we had hypothesized that 40% of patients in the control group would have a VAS score of 6 or more after the procedure (Lang et al., 2006), and in fact, only 14% scored ≥ 6. This is reflected in the analysis of the semi-structured interviews, where the patients mentioned the relief they felt immediately after the procedure, regardless of the study arm, whereas the apprehension before the procedure created much anxiety for them. Perhaps the timepoint at which anxiety was measured was too late for an accurate assessment of anxiety during the procedure. We wanted the measure of VAS anxiety to be taken as close as possible to the marker placement. Consequently, it was performed in the examination room by the radiological technicians who communicated with the patient using the CH technique (or not, according to the treatment allocation). To further reduce bias, even though the treatment allocation was not revealed to the patient, it might have been better if the VAS measure of anxiety had been done in the waiting room, as for the STAI Y-A score, and by another, independent person. The choice of anxiety measurement times is often tricky for this type of study, where one wishes to be as close as possible to the event without disturbing its progress.

In designing the study, the key point was to reduce the anxiety induced by the placement of the marker itself and therefore to demonstrate less anxiety with CH after the marker was placed. From the results, it seems that patients were more anxious before the wire was placed, and after the wire was placed, they felt relieved. It therefore would make more sense to focus an intervention on preparation for this procedure to reduce stress before and during the procedure. CH may therefore not be appropriate in this indication as it is not possible in practical terms for the radiology technician to perform CH prior to marker placement. It might be interesting to evaluate the effectiveness of self-hypnosis or anchoring in this indication, to empower the patient to be an actor in their treatment and to regain control of their symptoms and stress.

No specific method was used to ensure that the technicians delivered the CH as intended, since all of them were trained in CH and were used to performing CH in their daily practice before this study. We sought to reduce cross-contamination bias by stipulating that patients in the control group should be managed by radiological technicians who were not trained in CH. However, due to organizational constraints, almost one third of the patients in the control group had their procedure with a technician trained in CH. More generally, all the technicians and radiologists in the participating centres had been alerted to the potential benefit of CH, and thus, of the importance of creating a patient-career relationship based on dialog and trust [27]. The standard management was therefore likely to have been heterogeneous, since some radiologists and/or technicians may have utilized communication strategies similar to CH during their interactions with the patient (caring tone, non-aggressive vocabulary, explanations given before and during the procedure). The benefit of the CH technique may thus have been felt in both arms, leading to a potential differential classification bias in favor of the control group. Similarly, the way the patients were welcomed and prepared for the procedure was not supposed to be affected by the randomization group, i.e., a patient from the control group could be welcomed by a caregiver trained in hypnosis. This was obvious from the discourse of the interviews, where the patients from both groups frequently mentioned the climate of trust. They also reported that the factors that contributed to making them feel reassured during the procedure included the presence of other people in the room, smiles, and the interactions with the caregivers. Our results are in line with a previous study that aimed to evaluate the impact of CH on anxiety pre-and post-surgery in patients undergoing gynecological surgery (Sourzac et al., 2018). This study found relatively low levels of anxiety pre-surgery, linked to the use of CH upon reception at the hospital, which was determinant in creating a climate of trust. The authors underlined that the use of CH, by modifying the usual practices and the work environment, gradually brought more benevolence and serenity to the career-patient relations.

## Conclusion

This study failed to show a benefit of conversational hypnosis on anxiety in patients undergoing marker placement prior to surgery for breast cancer. The levels of anxiety in these patients before and after the procedure were comparable, and declined in a similar manner in both groups. Although two-thirds of patients in the control group underwent their procedure with a radiological technician who was not trained in CH, the fact that some caregivers had learned this personalized therapeutic communication technique may have had a positive impact on the whole caregiving team, since the patients from both groups reported feeling reassured thanks to the presence of caregivers in the room, and the smiles and benevolent communication of all the staff.

## Data availability statement

The raw data supporting the conclusions of this article will be made available by the authors, without undue reservation.

## Ethics statement

The studies involving human participants were reviewed and approved by the Ethics Committee CPP (Comité de Protection des Personnes) Est III on 3 May 2016 under the number ID-RCB: 2016-A00232-49, and by the French Agency for Health Products Safety (ANSM) on 22 April 2016 under the number 160225B-31. The patients/participants provided their written informed consent to participate in this study.

## Author contributions

LL, XG, PT, CH-S, and PH made substantial contributions to the conception, interpreted the patient data and drafted the work. VA and RE made substantial contributions to the conception, performed the qualitative analysis of the data and were a contributor in writing the manuscript. PS, AC, JG-D, JO, IK, and PW have made substantial contributions to acquisition of data. JS made substantial contributions to the conception, analyzed the data and was a contributor in writing the manuscript. J-LM contributed to editing the manuscript. All authors approved the final manuscript and agreed both to be personally accountable for the author’s own contributions and to ensure that questions related to the accuracy or integrity of any part of the work, even ones in which the author was not personally involved, are appropriately investigated, resolved, and the resolution documented in the literature. All authors contributed to the article and approved the submitted version.

## Funding

This study received the annual prize from the SFR-AFPPE in 2015 and was financed by the National Hospital Nurse and Paramedical Research Programme (Programme Hospitalier de Recherche Infirmière et Paramédicale—PHRIP; grant obtained in 2015).

## Conflict of interest

The authors declare that the research was conducted in the absence of any commercial or financial relationships that could be construed as a potential conflict of interest.

## Publisher’s note

All claims expressed in this article are solely those of the authors and do not necessarily represent those of their affiliated organizations, or those of the publisher, the editors and the reviewers. Any product that may be evaluated in this article, or claim that may be made by its manufacturer, is not guaranteed or endorsed by the publisher.

## Footnotes

^1^ http://www.tropes.fr/
